# Registered report protocol: Quantitative analysis of septin Cdc10-associated proteome in *Cryptococcus neoformans*

**DOI:** 10.1371/journal.pone.0242381

**Published:** 2020-12-14

**Authors:** Stephani Martinez Barrera, Stephanie Byrum, Samuel G. Mackintosh, Lukasz Kozubowski

**Affiliations:** 1 Department of Genetics and Biochemistry, Eukaryotic Pathogens Innovation Center, Clemson University, Clemson, SC, United States of America; 2 Department of Biochemistry and Molecular Biology, University of Arkansas for Medical Sciences, Little Rock, AR, United States of America; University of Michigan Health System, UNITED STATES

## Abstract

*Cryptococcus neoformans* is a pathogenic basidiomycetous yeast that primarily infects immunocompromised individuals. *C*. *neoformans* can thrive during infections due to its three main virulence-related characteristics: the ability to grow at host temperature (37°C), formation of carbohydrate capsule, and its ability to produce melanin. *C*. *neoformans* strains lacking septin proteins Cdc3 or Cdc12 are viable at 25°C; however, they fail to proliferate at 37°C and are avirulent in the murine model of infection. The basis of septin contribution to growth at host temperature remains unknown. Septins are a family of conserved filament-forming GTPases with roles in cytokinesis and morphogenesis. In the model organism *Saccharomyces cerevisiae* septins are essential. *S*. *cerevisiae* septins form a higher order complex at the mother-bud neck to scaffold over 80 proteins, including those involved in cell wall organization, cell polarity, and cell cycle control. In *C*. *neoformans*, septins also form a complex at the mother-bud neck but the septin interacting proteome in this species remains largely unknown. Moreover, it remains possible that septins play other roles important for high temperature stress that are independent of their established role in cytokinesis. Therefore, we propose to perform a global analysis of septin Cdc10 binding partners in *C*. *neoformans*, including those that are specific to high temperature stress. This analysis will shed light on the underlying mechanism of survival of this pathogenic yeast during infection and can potentially lead to the discovery of novel drug targets.

## Introduction

*C*. *neoformans* is a mycosis-causing opportunistic fungal pathogen, which is responsible for approximately 220,000 cases of cryptococcal meningitis, resulting in 180,000 global deaths annually [1]. Immunocompromised individuals, HIV patients, recent organ transplant recipients, and other patients that receive immunosuppressing drugs are susceptible to cryptococcal infection [2–4]. *C*. *neoformans* can reproduce both asexually, in the form of budding yeast, and sexually, which leads to production of spores that may act, in addition to desiccated yeast, as infectious propagules [5,6]. During initial stages of infection, after inhaled cells or spores reach the lungs, *C*. *neoformans* employs antiphagocytic factors such as capsule, melanin, and cell size enlargement, to evade the alveolar macrophages [7–9]. On the other hand, *C*. *neoformans* is capable of proliferating within the phagolysosome [9,10], an ability it utilizes as a Trojan horse mechanism to disseminate into the central nervous system (CNS) [11]. The ability to adapt and survive at 37°C is central to *C*. *neoformans* virulence [12–17]. Understanding the mechanisms that *C*. *neoformans* employs to thrive at host temperature is a potential route for deciphering novel drug targets for anti-cryptococcal therapy. *C*. *neoformans* septin proteins are a point of interest since they are essential for successful cytokinesis and survival at host temperature (37°C), while being dispensable for cytokinesis at 25°C [18].

Septins are a family of GTP-binding, filament-forming proteins that is conserved in eukaryotic cells, with the exception of plants [19–21]. Septins have been recently recognized as a novel component of the cytoskeleton; they assemble at the site of cell division and form a ring-like complex that contributes to cytokinesis [22]. Septins assemble into hetero-oligomeric complexes that form higher-order structures such as filaments, gauzes, and rings [21,23]. Septins were first identified in the baker’s yeast, *S*. *cerevisiae*, as crucial factors for the separation of the daughter from the mother cell in mitotically proliferating cells [23–25]. In proliferating *S*. *cerevisiae* cells, five septin proteins Cdc3, Cdc10, Cdc11, Cdc12 and Shs1 (which is most similar to Cdc11) comprise an array of filaments that is associated with the cell cortex at the mother–bud neck and controls cell polarity, bud morphogenesis, and cytokinesis [23,26]. *C*. *neoformans* encodes homologues of the following four core septins initially described in *S*. *cerevisiae*: Cdc3 (42.31% identity with *S*. *cerevisiae* Cdc3), Cdc10 (55.96% identity with *S*. *cerevisiae* Cdc10), Cdc11 (42.11% identity with *S*. *cerevisiae* Cdc11), and Cdc12 (48.91% identity with *S*. *cerevisiae* Cdc12) [18,20].

Septin proteins have been shown to associate with negatively charged membrane surfaces and have affinity for binding to a variety of phospholipids, specifically phosphoinositide [27]. It has also been determined that membrane association promotes septin assembly into filaments; therefore, the association of septins with membranes might be facilitated by the assembly of septins into higher-order structures [28]. Septins also associate with positively charged membranes and preferentially assemble on a positive membrane curvature [29,30]. This capability is a staple of proteins involved in intracellular membrane trafficking [29]. During the past decade, the following novel septin roles have been elucidated in mammalian cells: interacting with components of endocytosis and exocytosis [31,32]; acting as membrane diffusion barriers [33,34]; interacting with proteins involved in cytoskeleton organization [35,36]; interacting with proteins that are functionally associated with the ubiquitin and sumoylation cycles [35,37]. In addition, *S*. *cerevisiae* septins were recently implicated in autophagy [30,38]. Thus, septins in *C*. *neoformans* are likely involved in multiple cellular pathways through mechanisms that remain poorly characterized. The main goal of this study is to fill this knowledge gap by defining septin-interacting proteome.

## The rationale and design of the study

**Fig 1:**
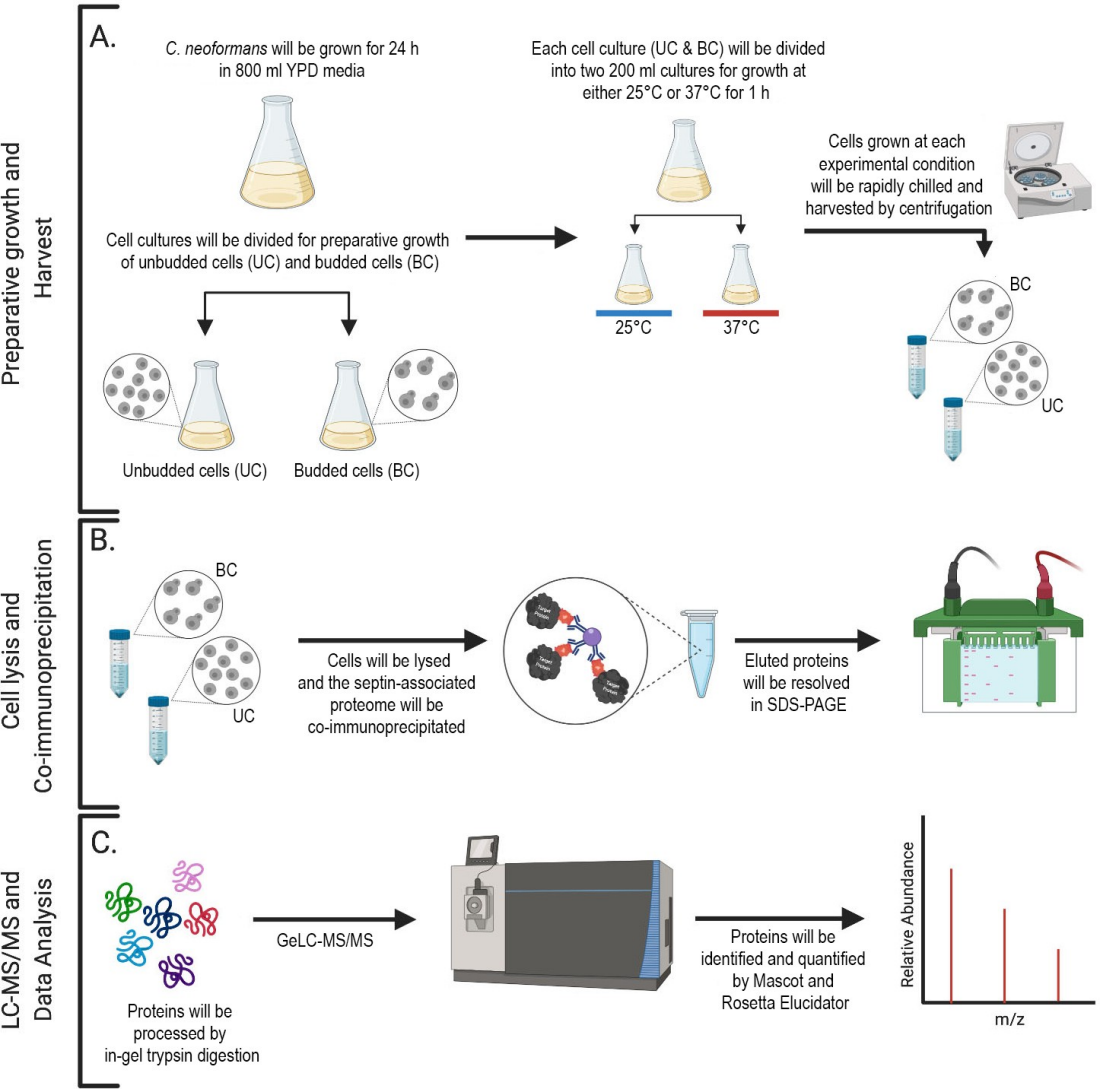
Schematic representation of GeLC-MS/MS workflow. (A) *C*. *neoformans* cells will be grown for 24 h in 800 ml YPD media at 25°C (2% yeast extract, 1% bacto-peptone, 2% dextrose, 2% bacto-agar). Subsequently, the cell culture will be split and half of the culture (400 ml) will be further grown in the same media for additional 48 h at 25°C (to assure it mostly consists of unbudded cells (UC)), while the other half will be refreshed in YPD media and grown for 3 h at 25°C (to assure it consists mostly of budded cells (BC)). The UC culture (after a total of 72 h incubation) will be refreshed for 30 min in 400 ml YPD at 25°C (to initiate the cell cycle). Each culture (the UC and BC) will be then divided into two 200 ml cell cultures (with cell densities ~ 10^7^ cells/ml) and grown either at 25 or at 37°C for 1 h prior to harvesting the cells. (B) Cell lysis and immunoprecipitation of bait protein using anti-mCherry antibodies. Subsequently, bound proteins will be eluted and resolved on SDS-PAGE gel. (C) Each lane of the SDS-PAGE gel will be excised into twelve pieces and subjected to in-gel trypsin digestion and analyzed by liquid chromatography tandem mass spectrometry (GeLC-MS/MS) on an Orbitrap Fusion Tribrid mass spectrometer (Thermo). Raw data will be processed with Mascot and Rosetta Elucidator to identify and quantify proteins.

A previous global quantitative analysis of septin Cdc11 interaction proteome in *S*. *cerevisiae* revealed 83 interacting partners in pathways such as ribosomal biogenesis, cell-cycle, and endocytosis [39]. Among Cdc11-interacting proteins, the remaining four vegetative septins were also detected. Thus, the Cdc11-interacting proteome detected by Renz C. et al. represents proteins that bind to the entire septin complex [40]. This is consistent with other studies that have shown that immunoprecipitation of Cdc3 or Cdc12 leads to purification of the entire septin complex [40,41]. The *C*. *neoformans* septin interactome has not yet been thoroughly analyzed for potential interacting partners. In contrast to *S*. *cerevisiae*, septin-dependent pathways are not necessary for viability at room temperature in *C*. *neoformans*. Yet, they become essential at higher temperatures or during other types of stress [18]. Thus, septins may play unique stress-related functions in *C*. *neoformans* that may be also conserved in other eukaryotes. In this study, tandem-mass spectrometry will be utilized to identify proteins that associate with septin complex in *C*. *neoformans* (Fig 1). The main aim of this study is to gain deeper understanding of septin function and septin structure assembly during nutrient rich growth at 25°C and temperature stress response at 37°C by identifying proteins that associate with septins. According to literature based on the *S*. *cerevisiae* model and a study performed in *C*. *neoformans*, septins in *C*. *neoformans* function as a complex that consists of all four septin proteins present in *C*. *neoformans* genome (Cdc3, Cdc10, Cdc11, and Cdc12). While *S*. *cerevisiae* Cdc10 is a part of the septin complex, it is not essential for its assembly at the mother-bud neck, although it plays a role in septin complex reorganization during cytokinesis [42]. Consistently, while the *C*. *neoformans* Cdc10 is dispensable for growth at 37°C, the remaining three septins are essential for growth at host temperature [18]. Furthermore, septin complex does not form in the absence of Cdc3 or Cdc12 [18]. Since Cdc10 is presumably dispensable for septin complex formation, we have designated it as “bait” for our pull down as tagging this protein should have a minimal impact on septin complex functionality. Our experimental approach will be also applied to determine which are the binding partners of septin Cdc10 in the absence of Cdc3, conditions at which functional higher order septin complex does not form [18]. Thus, our approach will also allow for identification of Cdc10 interactions that are independent of the formation of the whole septin complex. Such interactions may represent non-specific interactions and serve as a control or may indicate interactions that have physiological meaning yet have not been reported previously.

We hypothesize that cell cycle-related proteins will be among the candidates that will be identified in this study, given the conserved localization of septins in *C*. *neoformans* to the site of cell division [18]. On the other hand, we anticipate our analysis will reveal differences in the Cdc10 binding partners between *S*. *cerevisiae* and *C*. *neoformans* due to the following reasons: (1) septin proteins are essential for growth in *S*. *cerevisiae* at non-stress conditions, while they are dispensable for growth in *C*. *neoformans* only at non-stress conditions; (2) there is a significant phylogenetic divergence between these two species. Studies in other organisms also point to roles for septins that are independent of their established function during cytokinesis [35,39,43], and we anticipate our findings may reveal septin interactions supporting this possibility. Specifically, we will utilize samples of cells grown initially to stationary phase of growth (unbudded cells) and subsequently grown in refreshed media for 1.5 h, which is a time sufficiently long to allow restart of the cell cycle without substantial formation of budded cells in the population. Unbudded cells are expected to represent interactions with septins that are not relevant to cytokinesis.

## Materials and methods

### *C*. *neoformans* strains

All *C*. *neoformans* strains that will be included in this study were derived from *C*. *neoformans* wild type reference strain H99(*MAT*⍺) and are listed in Table 1. The strain expressing Cdc10-mCherry (LK60), and the *cdc3*Δ mutant expressing Cdc10-mCherry (LK160) were generated as previously described [18]. As two negative controls, H99 strain [17] and a strain that over-expresses mCherry ectopically will be generated via biolistic transformation. Primers will be designed to amplify the region of plasmid YSBE 596 (kindly provided by the Yong-Sun Bahn Lab) that contains an mCherry-encoding sequence flanked by a Hog1 terminator and a NAT resistance gene. Next, the amplified mCherry-NAT cassette will be introduced by biolistic transformation. The positive transformants will be confirmed by PCR and microscopy examination. For microscopy, cells will be grown overnight on YPD medium (2% yeast extract, 1% bacto-peptone, 2% dextrose, 2% bacto-agar) at room temperature, and refreshed the following morning using a 1:10 dilution. Cells will then be counted and diluted to achieve a concentration of 1×10^6^ cells/ml. Subsequently, 200–400 μl of cell suspension will be aliquoted into a microslide glass chamber (μ-Slide 2 Well Glass Bottom; Ibidi-cells in focus) for imaging. Brightfield, differential interface microscopy (DIC), and fluorescence images will be captured with Leica GDSF/TIRF inverted microscope. Images will be processed through ImageJ; Fiji software [44,45].

**Table 1:** Strains to be utilized in this study.

Strain	Genotype	Source/Reference
H99	(⍺) WT	(Perfect, et al. 1993) [17]
LK60	(⍺) *CDC10-mCherry*:*NEO*	(Kozubowski & Heitman, 2010) [18]
LK160	(⍺) *CDC3*::*NAT CDC10-mCherry*:*NEO*	(Kozubowski & Heitman, 2010) [18]
SM1	(⍺) *mCherry*:*NAT*	This study

### Antibody coupling and equilibration of NHS- magnetic sepharose beads for immunoprecipitation

NHS-Magnetic Sepharose (Millipore Sigma, Sigma-Aldrich) slurry will be mixed by vortexing, and 25 μl of medium slurry will be dispensed into a 1.5 ml microcentrifuge tube. Subsequently, the microcentrifuge tube will be placed in a magnetic rack to attract the beads. This technique will be employed for attracting the beads before every liquid removal step hereafter. At the same time, an anti-mCherry antibody solution will be prepared by diluting the anti-mCherry antibody 1mg/ml (Novus Biologicals) in coupling buffer (150 mM triethanolamine, 500 mM NaCl, pH 8.3 200 mM NaHCO_3_, 500 mM NaCl, pH 8.3). Then, the storage solution will be removed by pipetting. The Mag-Sepharose beads will then be washed once with 500 μl of ice-cold equilibration buffer (1 mM HCl). Immediately after equilibration, the Mag-Sepharose beads will be re-suspended by adding 50 μl of anti-mCherry antibody solution and then incubated for 30 min with slow end-over-end mixing at 4°C. After the incubation, the liquid from the binding reaction will be removed, and the mCherry-Mag Sepharose slurry will be washed once with 500 μl of blocking buffer-A (500 mM ethanolamine, 500 mM NaCl, pH 8.3), and subsequently with 500 μl of blocking buffer-B (100 mM Na-acetate, 500 mM NaCl, pH 4.0). The liquid will be then removed, and the mCherry-Mag Sepharose slurry will be incubated in 500 μl of blocking buffer-A for 15 min with slow end-over-end mixing in order to block any residual active groups. Next, the mCherry conjugated magnetic beads slurry will be washed once with 500 μl of blocking buffer-B, followed by a single wash with 500 μl of blocking buffer-A. Once more, the magnetic slurry will be washed with 500 μl of blocking buffer-B. The conjugated magnetic bead slurry will then be equilibrated for binding with the target protein by removing any remnant of blocking buffer-B from the Eppendorf tube, and re-suspension in 500 μl binding buffer (TBS (50 mM Tris, 150 mM NaCl, pH 7.5)).

Alternatively, the commercially available RFP-TRAP affinity resin (ChromoTek®, also available in the form of magnetic beads) will be utilized for immunoprecipitation of Cdc10-mCherry. The RFP-TRAP beads are coupled with RFP Nanobody/VHH, which possesses a high affinity towards mCherry-tagged fluorescent bait proteins. A lack of antibody in the protocol in this case has the advantage of leaving no contamination of heavy or light polypeptide chains derived from the primary antibody bound to the immunoprecipitation beads in the elution fractions. Thus, the overall yield obtained from the co-immunoprecipitation of the Cdc10-mCherry using both mCherry-NHS Mag Sepharose beads and ChromoTek RFP-Trap® agarose beads will be compared and the methodology that achieves the highest yield will be applied for anti-mCherry immunoprecipitation & GeLC-MS/MS (Orbitrap Fusion).

### Media and growth conditions

Unless otherwise stated, *C*. *neoformans* strains will be routinely maintained in YPD medium (2% yeast extract, 1% bacto-peptone, 2% dextrose, 2% bacto-agar) at 25°C. *C*. *neoformans* strains LK60, LK160, H99, and SM1 will be grown for 24 h in 800 ml YPD media at 25°C. Next, each 800 ml culture will be divided into two 400 ml cultures. For analysis of unbudded cells (UC), one of the divided 400 ml cultures will continue to be incubated in the same medium for an additional 48 h at 25°C (to assure it mostly consists of unbudded cells (UC)). After the 48 h incubation, the UC culture from each strain will be refreshed for 30 min in 400 ml YPD at 25°C (to initiate the cell cycle). Then, the 400 ml UC culture from each strain culture will be divided into two 200 ml cultures (cell densities ~ 10^7^ cells/ml), and one culture will be incubated at 25°C and the other at 37°C. After a one-hour incubation, cell cultures will be rapidly chilled using dry ice and independently centrifuged at 4°C. Next, cells from each experimental condition will be re-suspended in 15 ml of ice-cold lysis buffer containing protease inhibitors (10 mM Tris/Cl pH  =  7.5, 150 mM NaCl, 13% (v/v) glycerol, 0.5 mM EDTA; supplemented with protease inhibitor tablets (Roche cOmplete™, Mini, EDTA-free, Protease Inhibitor Cocktail (Millipore Sigma, Sigma-Aldrich)) and 1 mM PMSF). The re-suspended cell pellets will be aliquoted in 1 ml microcentrifuge tubes with screwcaps and then be disrupted with ~500 μl of 0.5 mm glass beads (Sigma-Aldrich) in a Mini-Beadbeater. Samples will be homogenized at 4°C for 40 sec, followed by 1 min incubations on ice. This process will be repeated 5 times to ensure that sufficient level of cell disruption has been achieved (initially confirmed by direct microscopic observation). Afterwards, the lysate of disrupted cells from each condition will be centrifuged at 40,000 × g for 15 min at 4°C to remove cell debris. The supernatant will be subsequently collected and will be stored at −80°C until use.

For analysis of budded cells (BC), the other 400 ml culture derived from the 800 ml culture (grown 24 h at 25°C) from each strain will be refreshed in YPD media and grown for 3 hours at 25°C (to assure it consists mostly of budded cells (BC)). Next, each 400 ml culture will be divided into two 200 ml cultures (cell densities ~ 10^7^ cells/ml). One BC culture from each strain will be incubated at 25°C and the other at 37°C. After a 1 h incubation, cell cultures will be rapidly chilled using dry ice and independently centrifuged at 4°C. Next, cells from each experimental condition will be process as described above for the unbudded cells (UC) samples.

### Anti-mCherry immunoprecipitation & GeLC-MS/MS (Orbitrap fusion)

The lysate from each experimental condition will be thawed on ice. The relative protein expression levels in the lysate will be ascertained as a control as previously described [46]. For analysis of proteins associated with Cdc10-mCherry in every experimental condition, ∼15 ml of lysate will be incubated for 1 h with slow end-over-end mixing at 4°C with either 200 μl of mCherry-NHS Mag Sepharose slurry prepared according to the manufacturer's instructions as described above (Millipore Sigma, Sigma-Aldrich) or 200 μl of ChromoTek RFP-Trap® agarose bead slurry. After the 1 h incubation, the mCherry-NHS Mag Sepharose beads will be washed 3× with 500 μl of wash buffer (TBS with 2 M urea, pH 7.5). The alternative RFP-Trap beads will be washed with a buffer based on the manufacturer instructions (Chromotek).

An important question is how to collect the bead-bound proteins for the subsequent analysis. Two most commonly utilized protocols are a direct in-bead digestion and a method where the proteins are released from the beads, resolved in SDS-PAGE and extracted from individual slices of the polyacrylamide gel. The advantage of the first method is that it reduces sample handling and minimizes the risk of protein degradation and sample contamination. The in-bead digestion however is not well suitable for protocols that utilize the antibody for immunoprecipitation of the bait protein due to a high background of antibody that may interrupt downstream analysis. Additionally, even in case an alternative pull-down method is employed (in our case the RFP-TRAP) there still is a possibility of the high bait protein background in the analysis. Therefore, our protocol will employ the SDS-PAGE and a subsequent in-gel digestion to avoid these issues. We would like to note that the disadvantage of the in-gel digestion method is that it may hypothetically eliminate from the subsequent analysis the proteins that migrate on the SDS-PAGE gel identically to the antibody or the bait protein, as the parts of the polyacrylamide gel containing those proteins will not be subject to analysis.

After the final batch of the wash buffer will be removed, the mCherry-NHS Mag Sepharose beads (or the RFP-TRAP beads) will be resuspended in 100 μl of 2x electrophoresis sample buffer. The samples will then be boiled for 10 min at 95°C to dissociate the immunocomplexes from the beads. Then, the eluted fraction will be removed and collected from the beads using a magnetic rack. The eluted sample (supernatant) will then be resolved on SDS-PAGE gel and stained with Coomassie blue. Each SDS-PAGE gel lane will be sectioned into 12 segments of equal volume. Each segment will be subjected to in-gel trypsin digestion as follows [47]. Gel slices will be de-stained in 50% methanol (Fisher), 50 mM ammonium bicarbonate (Sigma-Aldrich), followed by reduction in 10 mM Tris [2-carboxyethyl] phosphine (Pierce) and alkylation in 50 mM iodoacetamide (Sigma-Aldrich). Gel slices will then be dehydrated in acetonitrile (Fisher), followed by addition of 100 ng porcine sequencing grade modified trypsin (Promega) in 50 mM ammonium bicarbonate (Sigma-Aldrich) and incubation at 37°C for 12–16 h. Peptide products will then be acidified in 0.1% formic acid (Pierce). Tryptic peptides will be separated by reverse phase XSelect CSH C18 2.5 μm resin (Waters) on an in-line 120 x 0.075 mm column using a nanoAcquity UPLC system (Waters). Peptides will be eluted using a 45 min gradient from 98:2 to 67:33 buffer A:B ratio. [Buffer A = 0.1% formic acid, 0.5% acetonitrile; buffer B = 0.1% formic acid, 99.9% acetonitrile.] Eluted peptides will be ionized by electrospray (2.2 kV) followed by MS/MS analysis using higher-energy collisional dissociation (HCD) on an Orbitrap Fusion Tribrid mass spectrometer (Thermo) in top-speed data-dependent mode. MS data will be acquired using the FTMS analyzer in profile mode at a resolution of 240,000 over a range of 375 to 1500 m/z. Following HCD activation, MS/MS data will be acquired using the ion trap analyzer in centroid mode and normal mass range with precursor mass-dependent normalized collision energy between 28.0 and 31.0 [48,49].

## Data analysis

Proteins will be identified by database search using Mascot (Matrix Science) against the *C*. *neoformans* H99 UniProtKB database (Proteome ID: UP000010091; database last modified: May 4, 2020 [50]) with a parent ion tolerance of 3 ppm and a fragment ion tolerance of 0.5 Da. Scaffold (Proteome Software) will be used to verify MS/MS based peptide and protein identifications. Peptide identifications will be accepted if they can be established with less than 1.0% false discovery by the Scaffold Local FDR algorithm. Protein identifications will be accepted if they can be established with less than 1.0% false discovery and contain at least 2 identified peptides. Protein probabilities will be assigned by the Protein Prophet algorithm [51].

The spectrum counting report function of Scaffold will be utilized for performing spectral counting data analysis as previously described [46]. The data will be imported to Rosetta Elucidator software for robust label-free under the curve quantification, which will perform quantitative analysis between the 25°C and 37°C experimental conditions. This approach will be employed to determine whether or not there is differential binding between these two experimental conditions for each binding category. The peptide false discovery rate that will be used is 1%. The following values will be calculated as the sum of the peak: p-values, protein quantification, and associated fold-changes. The mass spectrometry obtained from this experimental design will be deposited in the ProteomeXchange–PRIDE repository [52,53].

A total of 16 sample types will be collected for mass spectrometry analysis (Table 2) and each sample type will be represented by 3 biological replicates to facilitate quantitative analysis. The following comparisons will be performed in order to gain maximum information from the data that has biological significance: 1. 25°C vs. 37°C; 2. Cells expressing Cdc10-mCherry vs. H99 cells or cells expressing mCherry only (negative controls); 3. Cells expressing Cdc10-mCherry vs. cells expressing Cdc10-mCherry and lacking Cdc3 (lacking a functional septin complex); 4. Unbudded cells (UC) vs. budded cells (BC) (to establish potential Cdc10 interactions that are independent of cytokinesis).

**Table 2:** Samples to be collected for the subsequent MS analysis. Each sample will be collected in triplicate.

	Strain			
Experimental condition	H99	SM1	LK60	LK160
25°C, UC	#1	#2	#3	#4
25°C, BC	#5	#6	#7	#8
37°C, UC	#9	#10	#11	#12
37°C, BC	#13	#14	#15	#16

Most promising/interesting potential protein interactions with Cdc10 will be further confirmed by tagging the protein of interest with GFP and performing an immuno-precipitation with anti-mCherry and/or anti-GFP antibody and detecting the conjugated proteins via western blot analysis. Candidate proteins will be also localized and colocalization with Cdc10-mCherry will be tested in cells at 25°C and after the shift to 37°C.
